# Pyrosequencing identification of *Mycobacterium tuberculosis *W-Beijing

**DOI:** 10.1186/1756-0500-2-239

**Published:** 2009-12-02

**Authors:** Zoheira Djelouadji, Mireille Henry, Amine Bachtarzi, Nadège Foselle, Didier Raoult, Michel Drancourt

**Affiliations:** 1 Unité de Recherche sur les Maladies Infectieuses et Tropicales Emergentes, UMR CNRS 6236, IRD 3R198, Université de la Méditerranée, IFR 48, Faculté de Médecine, Marseille, France

## Abstract

**Background:**

The worldwide expanding *Mycobacterium tuberculosis *W-Beijing family is associated with treatment failure and relapse. Its identification currently relies on spoligotyping and conventional sequencing. We developed pyrosequencing as an alternative method for its identification.

**Findings:**

Pyrosequencing found a G/A substitution in the Rv0927c-pstS3 intergenic spacer and a RD105 deletion, identifying 8/104 *M. tuberculosis *isolates as W-Beijing isolates. In addition, pyrosequencing found a previously unreported TGC deletion in the Rv0927c gene of W-Beijing isolates. Total concordance was found between the pyrosequencing data and conventional sequencing, as well as reference molecular identification. Multispacer Sequence Typing assigned the W-Beijing isolates to the Asian lineage and the 96 non-W-Beijing isolates to the Euro-American lineage (P < 10^-5^). The W-Beijing isolates were all susceptible to streptomycin, rifampin, isoniazid, ethambutol, and pyrazinamide; no resistance-associated mutations were detected in these eight W-Beijing isolates. There were no statistically significant differences in the antibiotic susceptibility of W-Beijing and non-W-Beijing isolates (*p *= 0.2, X^2 ^test). Pyrosequencing correctly identified *M. tuberculosis *organisms in 26/26 sputum specimens exhibiting acid-fast bacilli. Pyrosequencing results were obtained within four hours, incurring an estimated cost of 1.86 €/test.

**Conclusion:**

Pyrosequencing of the Rv0927c gene and adjacent intergenic spacer is an efficient, low-cost technique for the rapid identification of W-Beijing isolates.

## Background

Tuberculosis is the leading cause of death due to a single infectious agent in adults, killing about three million people every year [1]. One-third of the human population is thought to be infected by *Mycobacterium tuberculosis*, the main bacterial agent of human tuberculosis [2]. DNA fingerprinting analyses discriminated several *M. tuberculosis *lineages [3,4], including the W-Beijing family initially found among Chinese isolates [5]. W-Beijing isolates were subsequently shown to be highly prevalent throughout Asia and to have spread to other continents and countries, including the USA and South Africa [6], exhibiting a prevalence as high as 62% in some geographic areas [7]. In addition to their worldwide diffusion, W-Beijing isolates induce a more severe pathology than other *M. tuberculosis *genotypes by eliciting a reduced level of cytokine and a lack of associated Th1-type immunity [8]. W-Beijing organisms have been also been linked to drug resistance, treatment failure and relapse [9,10].

These data emphasize the importance of rapid identification of W-Beijing isolates for epidemiology and infection control. W-Beijing isolates can be identified by spoligotyping, which reveals the absence of spacers 1-34 and the specific A1 insertion of an IS *6110 *element in the origin of replication [5]. Genomic microarray approaches revealed several large sequence polymorphisms which classified W-Beijing isolates into five subtypes [4,11]. In particular, RD105 was found to be specific to W-Beijing isolates [11]. Reverse genetics demonstrated one deletion in the Rv0927c gene and a single nucleotide polymorphism (SNP) in the Rv0927c-pstS3 intergenic spacer, both of which are specific to W-Beijing organisms [12]. However, time and cost as well as technical demand of these methods still limit their wide application in laboratories. Efforts towards the development of more rapid and affordable methods yielded the development of polymerase chain reaction-single-strand conformational polymorphism (PCR-SSCP) analysis [13].

Pyrosequencing technology is a sequencing method that is mostly used for short-read sequencing and SNP analyses. It has been used for the identification of various bacteria including mycobacteria [14], as well as for the detection of drug-resistance in *M. tuberculosis *[15-17].

In the present study, we developed an Rv0927c gene and adjacent intergenic spacer pyrosequencing method for the rapid identification of W-Beijing isolates, and applied this method to identify W-Beijing isolates among *M. tuberculosis *isolates made in our laboratory over two years.

## Findings

### Methods

#### Bacterial strains

A collection of 104 *M. tuberculosis *isolates cultured from respiratory tract specimens as part of the routine activity in our laboratory in 2005-2006 were identified by conventional methods [18]. For each isolate, one colony grown on 5% sheep blood agar (Biotechnology Appliquée, Dinan, France) was removed using a sterile loop, and mixed with freezing beads for storage at -20°C prior to inactivation and DNA extraction with a Qiagen kit (Qiagen, Courtaboeuf, France). This study has been approved by the Ethics Committee, Marseilles.

#### Conventional DNA sequencing, reference molecular identification, and Multispacer Sequence Typing (MS) genotyping

The Rv0927c gene and Rv0927c-pstS3 intergenic region were PCR amplified as previously described in the presence of negative controls consisting of PCR mix without DNA [12]. PCR products were sequenced by Sanger sequencing using the BigDye Terminator v1.1 Cycle Sequencing kit (Applied Biosystems, Courtaboeuf, France). Sequencing electrophoresis was performed with a 3100 genetic analyzer (Applied Biosystems) in both directions. The sequences were edited using the Auto assembler program (Applied Biosystems) and aligned using CLUSTAL W http://bioinfo.hku.hk/services/analyseq/cgi-bin/clustalw_in.pl and NPS Multalin multiple alignment http://npsa-pbil.ibcp.fr. *M. tuberculosis *isolates identified as belonging to the W-Beijing family were confirmed by parallel detection of the RD105 deletion as previously described [4]. *M. tuberculosis *isolates were also genotyped using MST to assess their phylogeographical lineage, as previously described [19].

#### Pyrosequencing

Based on polymorphisms identified using the above mentioned conventional DNA sequencing as previously described [12], the pyrosequencing primers used in this procedure were designed using the PSQ assay design (Biotage, Uppsala, Sweden) (Table 1). The Rv0927c intergenic region was amplified using Rv0927c-pstS3F. biotin and Rv0927c-pstS3R; PCRs were performed at a final volume of 50 μL containing 33 μL of H_2_O, 5 μL of 10× buffer (Qiagen), 2 μL of 25× MgCl_2_, 5 μL of 10× dDNTP, 1 μL of forward primer (10 pmol/μL), 1 μL of reverse primer (10 pmol/μL), 0.25 μL of Hotstart Taq (Qiagen), and 2 μL of target DNA. Appropriate negative controls consisting of PCR mix without target DNA were also included for each *M. tuberculosis *isolate. PCRs were performed according to the following program: enzyme activation at 95°C for 15 min, followed by 34 cycles comprising 95°C for 30 s, 60°C for 30 s, 72°C for 1 min, and a 5-min final elongation step at 72°C. The Rv0927c gene was amplified in parallel using Rv0927c F. biotin and Rv0927c R (Table 1), as described above. The expected 70-bp product was analyzed in a 2% agarose gel. Fifty microliters of binding buffer (Pyrosequencing AB, Uppsala, Sweden) and 5 μL of streptavidin-coated Sepharose beads were added to 50 μL of PCR product, and the solution was vigorously mixed for 10 min. The biotinylated PCR products were immobilized on streptavidin-coated Sepharose beads and separated from the nonbiotinylated strand by denaturation in 200 μL of denaturation buffer (Pyrosequencing AB) for 5 s, followed successively by vacuum filtration in 200 μL of ethanol, 200 μL of washing water, and 200 μL of sterile water. The templates were then transferred to a 96-well plate containing 43.5 μL of annealing solution and 1.5 μL of sequencing primer. The annealing reaction was performed at 83°C for 2 min on a thermoblock (Applied Biosystems). Pyrosequencing was performed with an automated PSQ™ 96 system (Pyrosequencing AB). The four different deoxynucleotide triphosphates were added, with cyclic dispensation of the enzymes and substrates (Pyrosequencing AB). The pyrosequencing data were evaluated using Peak Height Determination Software v1.1 (Pyrosequencing AB), and 96 reactions were completed in 11 min.

**Table 1:** Primers used for amplification and pyrosequencing analysis of Rv0927c in *M. tuberculosis *isolates.

Primer name	Sequence (5'-3')
RV0927c-pstS3F. biotin	Biotin-TTGACCCCTGATGATGGAC
RV0927c-pstS3R	ACGGCATACGGACATCCTT
RV0927c-pstS3Seq	ACATCCTTCCCCTGA
RV0927c F	CCGGAGTGTTCCAGCATCA
RV0927c R. biotin	Biotin-GGACGCCTTCGCCTTCAA
RV0927c Seq	CACCGCCGCGACGGTC

#### Antibiotic resistance analysis

The susceptibility to streptomycin, rifampin, isoniazid, ethambutol and pyrazinamide was tested according to standard methods [18]. Rifampin, isoniazid, and ethambutol-resistance were screened among *M. tuberculosis *isolates based on characterization of resistance-associated hot mutations, as previously described by pyrosequencing [15].

#### Statistical analyses

A correlation between *M. tuberculosis *isolates identified as belonging to the W-Beijing family and their assignment into a phylogeographical lineage defined by MST was tested using the Chi Square test (Epi Info version 3.4.1, Centers for Disease Control and Prevention, Atlanta, USA). We also evaluated differential susceptibility to antibiotics between W-Beijing and non-W-Beijing isolates.

#### Clinical specimens

We selected 26 respiratory tract specimens, which were positive by Ziehl-Neelsen staining, and cultured the identified *M. tuberculosis *using conventional methods [18]. Pyrosequencing was performed directly on inactivated clinical specimens. Eight negative controls consisted of respiratory tract specimens that were negative for Ziehl-Neelsen staining, and these controls remained negative in culture

## Results and Discussion

Rapid expansion of the *M. tuberculosis *W-Beijing family in various parts of the world urged the development of molecular tools for its rapid detection in the clinical laboratory. Current methods rely on PCR pattern analysis, but lack sequencing confirmation for the RD105 deletion method and PCR-SSCP method [13] or rely on conventional Sanger sequencing, a one-day long procedure that delays detection [11]. In the present study, we took advantage of the ease of pyrosequencing technology to develop a method for the rapid detection of W-Beijing isolates.

**Figure 1:**
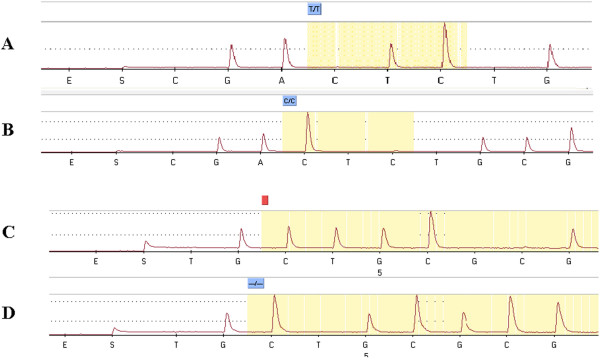
**Pyrosequencing chromatograph of the wild type Rv0927c-pstS3 intergenic region (A), the W-Beijing type Rv0927c-pstS3 intergenic region (B), the wild type Rv0927c gene (C), and the W-Beijing type Rv0927c gene (D)**.

In all PCR experiments, negative controls remained negative and *M. tuberculosis *isolates yielded an Rv0927c-pstS3 intergenic spacer amplicon of the expected size (270 bp) and Rv0927c gene fragment of 790 bp. Pyrosequencing patterns were clear and accurate, and the sequences obtained for codon 127 in the Rv0927c-pstS3 intergenic spacer, as well as a new deletion at codon 115 in the Rv0927c gene, could be read directly from the pyrosequencing spacer (figure 1). Eight of 104 isolates yielded the G/A substitution in the Rv0927c-pstS3 intergenic region, in contrast to the remaining 96 isolates (figure 1). The RD105 deletion was also detected in the same eight isolates (PCR product size, 761 bp instead of 1,466 bp in the remaining 96 isolates). These features identified the 8 isolates as W-Beijing isolates as confirmed by reference methods. Pyrosequencing assignment agreed with conventional DNA sequencing and reference molecular assignment for 104/104 (100%) *M. tuberculosis *isolates.

In the Rv0927c gene, pyrosequencing clearly identified a new TGC deletion in the eight W-Beijing isolates instead of the AGC deletion previously described [12]. We verified the absence of this new TGC deletion in complete genome sequences of *M. tuberculosis*, and BLAST analysis against the Rv0927c gene sequence indicated that this TGC deletion was original (figure 1). The Rv0927c gene sequence in the other 96 *M. tuberculosis *isolates showed complete identity with the reference non-W-Beijing *M. tuberculosis *H37Rv strain (Genbank: AL123456). This deleted sequence could still be translated *in silico*. MST identified the ST3 profile in 8/8 W-Beijing isolates, assigning them to the Asian lineage [19] (P < 10^-5^). The remaining 96/104 *M. tuberculosis *were distributed into 10 ST profiles and assigned to the Euro-American lineage (Table 2).

**Table 2:** Assignment of *M. tuberculosis *to a geographic lineage by MST.

Geographic lineage	MST profile	Number of isolates
Asian lineage	ST3	8
Euro-American lineage	ST8	29
	ST19	17
	ST17	13
	ST23	10
	ST6	8
	ST28	7
	ST14	5
	ST21	3
	ST8	2
	ST4	2

The fact that pyrosequencing found a previously unreported deletion in W-Beijing isolates illustrates the advantage of sequencing over non-sequencing-based methods of identification. Sequencing-based methods allow for the discovery of new genotypes within bacterial species, while latter methods could miss such variants and could yield inaccurate identification data. While sequencing is of interest for accurate identification of *M. tuberculosis *isolates, conventional Sangers'method is a time and resources-consuming method. In the present study, pyrosequencing was performed within five hours, including a four-hour PCR. The cost of a pyrosequencing reaction is ~1.86 €, less than our conventional DNA sequencing reaction, which is ~2.64 €. The objectivity of this technique is extremely useful for medical microbiology applications, as the skilled interpretation of bands required for RFLP [20] is not necessary. Finally, the ability to perform the pyrosequencing assay using inactivated clinical samples has been demonstrated previously [21] and provides a considerable advantage; for example, this high-throughput assay can prevent multi-drug resistant episodes via rapid screening of the W-Beijing isolates. In this study, PCR products were generated from all 26 respiratory tract, acid fast bacilli-positive clinical specimens, and negative controls remained negative. Pyrosequencing identified no polymorphisms in the Rv0927c-pstS3 intergenic spacer or Rv0927c gene, thereby identifying these organisms as non-W-Beijing; MST further assigned them to the Euro-American lineage [19].

The 7.7% prevalence of W-Beijing isolates we found in a large cosmopolitan area of Southern France is higher than that reported in other French areas [22] and in Western Europe [23]. The antibiotic susceptibility pattern of the 104 *M. tuberculosis *isolates was assessed by molecular and conventional methods. The eight W-Beijing isolates characterized herein were susceptible to 5/5 antibiotics tested, unlike those reported in other studies [24]. No statistically significant differences were observed regarding *in vitro *susceptibility between eight W-Beijing and 96 non-W-Beijing isolates (*p *= 0.2, X^2 ^test). Molecular analyses revealed no mutations associated with drug resistance in the eight W-Beijing isolates. However, there is no constant association between a W-Beijing family and drug resistance [25]. For example, the acquisition rate for *rpoB *gene point mutations conferring rifampin resistance is similar in W-Beijing and non-W-Beijing isolates [26].

## Conclusion

In conclusion, we developed a pyrosequencing-based method for rapid, clear, and accurate detection of W-Beijing *M. tuberculosis *isolates and respiratory tract specimens. In this study, W-Beijing isolates comprised 7.7% of *M. tuberculosis *and belonged to the East Asian lineage. The low-cost assay described herein could be used to complement current routinely used techniques, and could replace the time-consuming molecular tests presently used to identify W-Beijing isolates.

## Competing interests

The authors declare that they have no competing interests.

## Authors' contributions

ZD performed the experiments, analyzed the data, and wrote the manuscript. MH performed the experiments and analyzed the data. AB performed a portion of the experiments. NF performed a portion of experiments, DR obtained funding, and MD conceived of the study, analyzed the data, and wrote the manuscript. All authors read and approved the final manuscript.
